# Developing an Antenatal Intervention to Improve Vaccine Uptake Using Parent, Patient and Public Involvement and Engagement in Northern Ireland

**DOI:** 10.1111/hex.70795

**Published:** 2026-08-04

**Authors:** Stephanie Ann McCarron, Lynsey Patterson, Nigel Hart

**Affiliations:** ^1^ Queens University Belfast Belfast NorthernIreland

**Keywords:** antenatal vaccination, community engagement, Intervention development, maternal health, patient and public involvement, public engagement, vaccine uptake

## Abstract

**Introduction:**

Patient and public involvement and engagement (PPIE) is increasingly recognised as important in health care research, yet reporting of how involvement is undertaken and how it informs intervention development remains inconsistent. In maternal health, information about antenatal vaccination is required early in pregnancy, highlighting the value of involving parents and communities in shaping how such information is developed and shared. This paper describes the use of Parent, Patient and Public Involvement and Engagement (PPPIE) to inform the development of an intervention to support antenatal vaccine uptake in Northern Ireland (NI).

**Methods:**

Involvement and engagement activities were embedded during the intervention development phase and informed by established guidance for inclusive practice. Partnerships were formed with a Maternity Service Liaison Committee (MSLC), Sure Start programmes in areas of social disadvantage and an outreach antenatal clinic serving an East Timorese community. Engagement approaches were adapted to each setting and included facilitated discussions, written feedback and digital tools to support participation. Trusted intermediaries and translators were used where appropriate and activities were documented to support reflection and transparency.

**Results:**

Input from parents and community members informed the content, timing and presentation of antenatal vaccine information within the developing intervention. Contributors highlighted the importance of clear, plain language information early in pregnancy and the value of tailoring information to different needs. Working through established community networks supported engagement with groups who are often underrepresented in research. Feedback was shared with contributors using a ‘you said, we did’ approach to demonstrate how their input influenced the developing intervention.

**Conclusions:**

This paper provides a practical case study of how PPPIE can be embedded within antenatal intervention development. By describing approaches and learning, it contributes to the evidence base on involvement and engagement in maternal health research and offers insights for others seeking to undertake similar work in maternal health.

**Patient or Public Contribution:**

Parents and members of the public were involved through PPPIE activities undertaken during the design and development of the intervention. Their input informed the content, timing and presentation of antenatal vaccine information and supported refinement of the intervention during development.

**Abbreviations**
MSLC(s): Maternity Service Liaison Committee(s)
NI: Northern Ireland
NIHR: National Institute for Health and Care Research
NPEU: National Perinatal Epidemiology Unit
PHA: Public Health Agency
PPIE: Patient and Public Involvement and Engagement
PPPIE: Parent, Patient and Public Involvement and Engagement
PRU‐MNHC: Policy Research Unit in Maternal and Neonatal Health and Care
UK: United Kingdom

## 1 Introduction

Patient and Public Involvement and Engagement (PPIE) in research is increasingly recognised as essential. Despite its growing prominence, the reporting of PPIE remains inconsistent, limiting its broader applicability and utility in health research [1]. There is a pressing need to integrate the voices of affected populations earlier in the research process ‐ particularly those underrepresented in research, to ensure that developed interventions are both feasible and attuned to the needs of end users [2].

This is especially crucial in maternal and neonatal health, where expectant parents frequently navigate complex decisions impacting on both their health and that of their developing baby. This research draws upon established guidance for PPIE in maternal and neonatal health to support the shaping and delivery of an intervention aimed at enhancing information sharing, specifically around antenatal vaccination, during the earliest stages of pregnancy in NI's healthcare context [2, 3, 4, 5].

The proposed intervention seeks to improve information dissemination regarding antenatal vaccination, with the ultimate aim of supporting vaccine uptake and reducing vaccine preventable morbidity and mortality amongst mothers and infants. This paper focuses on the role of involvement and engagement in shaping the intervention, rather than on intervention effectiveness, which will be evaluated separately.

The United Kingdom's (UK) National Institute for Health and Care Research (NIHR) defines PPI as an active partnership between researchers and the public, working ‘with’ or ‘by’ people who use services, rather than ‘to,’ ‘about,’ or ‘for’ them [6]. In Northern Ireland, the Health and Social Care Research and Development Division similarly mandates the inclusion of service users and carers in the planning and delivery of health and social care services [7]. Together, these frameworks emphasise the importance of meaningful involvement throughout the research lifecycle.

Guidance on developing health interventions further underscores the value of collaboration with stakeholders from the target population [2]. In maternal health research, this includes women, parents and service users, alongside healthcare professionals. The National Perinatal Epidemiology Unit (NPEU) and its affiliated Policy Research Unit in Maternal and Neonatal Health and Care (PRU‐MNHC) have developed frameworks to guide Parent, Patient and Public Involvement and Engagement (PPPIE) within this context [3, 4, 5]. Their PPPIE strategy encompasses diverse contributors including mothers, fathers, partners and others affected by reproductive health policies and distinguishes between participation, involvement and engagement [3].

These principles underpin the PPPIE approach described in this paper [3]. We describe how PPPIE was embedded within the development of an antenatal vaccination intervention in NI and reflect on learning from this process to inform future maternal health research and practice.

## 2 Methods

### 2.1 Study Design and Approach

This paper reports on PPPIE activities undertaken to inform the development of an antenatal vaccination intervention in Northern Ireland. The planning and delivery of PPPIE activities were informed by recognised frameworks and reporting guidance. The PPPIE strategy developed by the PRU‐MNHC provided overarching principles to guide inclusive involvement with parents and families in maternal health research [3]. Practical tools within this strategy, including the ‘8 R's’ framework, supported structured reflection on reach, relevance, refinement and relationships, throughout the process. This framework was used as a monitoring tool throughout PPPIE delivery, supporting structured reflection on who was engaged (reach), how well engagement aligned with intervention development aims (relevance), how learning informed iterative changes (refinement) and how trust and ongoing engagement were supported across settings (relationships) [3]. Reflections were documented contemporaneously and reviewed by the team to inform ongoing adaption of engagement approaches and intervention materials.

The GRIPP2 reporting checklist was used to support transparent documentation of involvement activities and learning [1]. Additional guidance from Oxford Population Health Listening Series was used to strengthen inclusivity, particularly when engaging with communities experiencing social disadvantage or health inequalities [8]. In practice, this included: 1) building trust by working through established relationships and referral pathways via maternity and community stakeholders (e.g. Links from senior leadership to MSLC contacts and use of trusted intermediaries within Sure Start and local midwifery networks; 2) involving underrepresented voices as early as possible by sharing an early visual prototype (the antenatal timeline) for feedback prior to formal design work; 3) using clear, audience appropriate communication materials (visual aids/infographics and plain‐language information) and actively inviting feedback on clarity and format; and 4) demonstrating impact by feeding PPPIE learning into internal stakeholder discussions to progress intervention development and by sharing ‘you said, we did’ feedback through original engagement channels to show how input shaped outputs.

### 2.2 Settings and Participants

PPPIE contributors were recruited through established community and service‐based settings via Sure Starts. Sure Start is a community based early years and family support programme in the UK (including Northern Ireland), providing services for parents and young children, with local programmes often focusing on families in areas of social disadvantage. We engaged 1) one service user representative attending a Maternity Service Liaison Committee (MSLC) meeting, 2) expectant parents attending antenatal classes delivered within Sure Start programmes (Dungannon/Coalisland and Clogher Valley, typically approximately 6–10 expectant couples per session) and 3) expectant parents attending an outreach antenatal clinic serving the East Timorese community in Dungannon (10 one‐to‐one conversations over three clinic sessions over 3 days). In addition, broader public input was sought via an MSLC‐associated social media forum (NI Maternity Forum, operated by Birth Wise charity, with approximately 3,500 members), through which 10 anonymised comments were relayed to the study team.

We did not collect formal demographic data (e.g. Ethnicity/nationality) from PPPIE contributors as part of these involvement activities. However, settings were purposively selected to facilitate engagement with groups who may be underrepresented in research, including families accessing Sure Start services in areas of social disadvantage and expectant parents attending an outreach clinic serving the East Timorese community. Where required, community‐based translation support was used to ensure discussions were accessible and culturally appropriate.

### 2.3 PPPIE Activities and Data Collection

Engagement methods were tailored to each setting and designed to be flexible and accessible. Activities included facilitated group sessions during antenatal classes, one‐to‐one conversations in clinic settings and opportunities for written or anonymous digital feedback. A combination of visual prompts, printed materials and digital tools including interactive polling software, was used to support participation and encourage discussion. In addition to in‐person engagement, broader engagement was facilitated through a social media platform associated with a MSLC [9]. This enabled wider input from parents and service users and facilitated engagement beyond formal group settings.

By using existing relationships with community‐based and maternity services, engagement was facilitated by the involvement of familiar professionals and facilitators supporting open discussion and feedback. Native language translation support was used within the outreach antenatal clinic to enable meaningful participation and to ensure discussions were accessible and culturally appropriate. Attention to language, format and delivery of engagement activities helped support inclusive involvement, particularly when engaging families accessing services in areas of social disadvantage and with expectant parents attending an outreach clinic serving an ethnic minority community.

Feedback focused on parents experiences of receiving antenatal information, perceived gaps in timing or content and preferences for how information should be presented within early pregnancy care pathways. Contributions were documented contemporaneously using digital tools and spreadsheets to support transparency and reflection. Learning from involvement activities was reviewed regularly and used to inform iterative refinement of the developing intervention.

### 2.4 Intervention Development and Design Process

The initial intervention concept originated from the lead researcher and was informed by: 1) an image within the NICE routine antenatal care guidance depicting the recommended schedule for routine antenatal care (including differences by parity) [10], and; 2) findings from a prior scoping review indicating that pregnant women wanted clear, accessible vaccination information presented in an easy to understand format [11]. The proposed intervention aimed to adapt the NICE antenatal care timeline to show when vaccinations are recommended in pregnancy and to provide signposting to further reliable information. The concept was discussed with senior leadership within the Public Health Agency (PHA), whose role it is to protect and improve the health and social wellbeing of the Northern Ireland population, who endorsed the intervention concept allowing PPPIE to commence.

**Figure 1:**
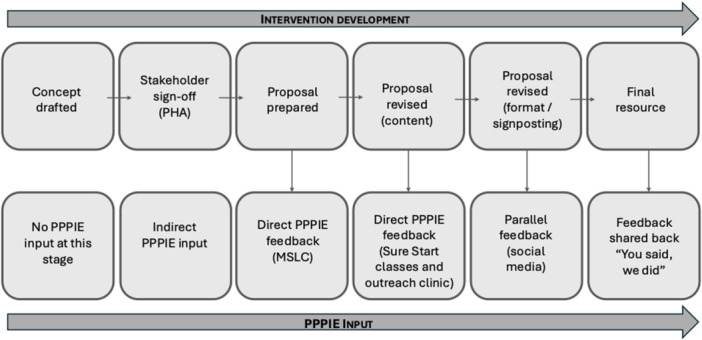
Overview of intervention development stages and PPPIE involvement.

PPPIE contributors also endorsed the concept, and highlighted the importance of timely provision of reliable, trustworthy information early in pregnancy and supported the proposed timing of delivery. Feedback shaped implementation features, including preference for a digital, interactive format, resulting in the inclusion of QR codes and clickable hyperlinks to further information. Contributors suggested addition of early pregnancy‐relevant information, leading to inclusion of signposting to wider pregnancy resources (e.g. early pregnancy information resources and self‐care guidance). Contributors also proposed further enhancements (e.g. multiple language formats and app based resources) which were not implemented within the scope, funding and practical constraints of this work. Figure 1 provides a visual overview of the this process of intervention development and PPPIE involvement. A fuller account of PPPIE outputs and learning will be reported separately in a dedicated PPPIE outcomes paper.

### 2.5 Ethical Considerations

Formal research ethics committee approval was not required for this work, as the activities described constitute involvement and engagement undertaken to inform intervention development rather than research involving the collection of personal data. Ethical considerations were nonetheless central to the design and delivery of all PPPIE activities and guided by published best practice for ethical involvement.

Participants were provided with clear information about the purpose of involvement activities and how their contributions would be used. Participation was voluntary, and individuals were free to decline or withdraw at any point without consequence. Safeguards were implemented to minimise potential distress, including the presence of trusted intermediaries and the option to engage anonymously where appropriate. Participants were offered refreshments and reimbursement in line with the nature and duration of involvement activities, consistent with published guidance on appropriate and ethical recognition of their time [3, 8]. Reimbursement was proportionate and tailored to the context of each engagement setting.

## 3 Results

### 3.1 Overview of PPPIE Contributors and Reach

PPPIE activities engaged expectant parents and service users across a range of community and service‐based settings, including Sure Start programmes (Dungannon/Coalisland and Clogher Valley), an MSLC meeting (including service user representation) and an outreach antenatal clinic serving the East Timorese community in Dungannon. Engagement occurred through facilitated group discussion within antenatal classes, one‐to‐one conversations within clinic settings and broader public input via an MSLC‐associated social media forum.

### 3.2 Learning from PPPIE: Shaping the Intervention

Across engagement activities, contributors consistently emphasised the importance of receiving clear, plain language information about antenatal vaccination early in pregnancy. Participants described uncertainty regarding which vaccines are recommended, the timing of vaccination and how vaccination protects both mothers and babies. Variability in how and when information was accessed across care pathways was also highlighted.

Input from PPPIE contributors directly informed the content and structure of the developing intervention. Feedback supported the inclusion of concise, visual information explaining what vaccines are offered in pregnancy and when they are recommended. Contributors also highlighted the need for information to be accessible in multiple formats and adaptable to individual preferences.

Engagement with parents reinforced the value of positioning antenatal vaccine information at the point of antenatal self‐referral, rather than later in pregnancy, to support informed decision‐making at an early stage.

### 3.3 Outputs and Resources Developed

PPPIE input informed the development of a revised antenatal timeline figure incorporating recommended vaccination timing in pregnancy, supported by brief plain‐language text. The resource was adapted to a digital format and included QR codes and clickable hyperlinks to signpost users to further trusted information. In response to feedback suggesting broader early pregnancy support, additional signposting to pregnancy relevant resources was incorporated alongside vaccine information.

### 3.4 Monitoring, Feedback and Refinement

PPPIE activities were monitored and documented using the 8 R's framework [3], supporting structured reflection throughout intervention development. Learning from PPPIE activities informed iterative refinement of the intervention, including incorporation of additional pregnancy‐relevant information in response to contributor feedback and the inclusion of QR codes and hyperlinks to signpost users to further information. Feedback to contributors was provided using a ‘you said, we did’ approach through original engagement channels, demonstrating how involvement influenced the developing intervention and supporting transparency and accountability within the PPPIE process.

## 4 Discussion

### 4.1 Principal Findings

This paper describes how PPPIE was embedded within the development of an antenatal vaccination intervention in Northern Ireland. PPPIE informed the timing, content and presentation of antenatal vaccine information, with contributors consistently emphasising the importance of clear, accessible information early in pregnancy. Engagement across community and service based settings (including services supporting families experiencing social disadvantage and an outreach clinic serving an ethnic minority community) helped ensure the intervention was shaped by real‐world information needs. By focusing on PPPIE during the intervention development phase, this work provides practical insight into how involvement and engagement can be operationalised within maternal health research and used to meaningfully shape intervention design.

### 4.2 PPPIE in Early Pregnancy Intervention Development

Much of the existing literature on antenatal vaccination interventions focuses on delivery after the earliest stages of pregnancy, often within routine clinical encounters. In contrast, this work highlights the value of using PPPIE to inform the development of antenatal vaccine information that is positioned earlier in the maternity care pathway, at a point when parents are actively seeking information and forming expectations about pregnancy care. Contributors described gaps in the timing and accessibility of antenatal vaccine information, reinforcing findings from previous research that emphasise the need for timely, comprehensible information to support informed decision making in pregnancy [11]. Involving parents and communities during early intervention development enabled the intervention to be shaped around information needs identified through PPPIE, rather than assumptions held by researchers or healthcare professionals. This approach supports the growing recognition that intervention acceptability and feasibility are strengthened when end users are involved early and iteratively, particularly in areas such as maternal vaccination where trust, understanding and timing are central to decision‐making [11].

### 4.3 Inclusivity, Trust and Setting

The PPPIE process reflected key principles associated with inclusive involvement, including trust, communication and empathy, as articulated within the Oxford Population Health Listening Series [8]. Our experience supports the view that inclusive involvement is not achieved solely through recruitment strategies but through attention to context, relationships and how engagement is facilitated (including culturally sensitive approaches and translation support where needed) [3, 8]. These considerations were particularly relevant when engaging with parents accessing services in areas of social disadvantage and with expectant parents attending an outreach clinic serving an ethnic minority community.

### 4.4 Use of the 8 R's Framework in PPPIE Monitoring

The 8 R's framework provided a useful structure for monitoring and reflecting on PPPIE activities [3]. In this paper, it supported a pragmatic approach to documenting and reviewing PPPIE learning during intervention development, illustrating the value of applying PPPIE frameworks flexibly and proportionately to fit the context and purpose of the work.

### 4.5 Strengths and Limitations

A key strength of this work is the embedding of PPPIE across multiple settings, enabling engagement with parents and service users through community and maternity services, including settings that may support groups who may be underrepresented in research. This paper focuses on PPPIE undertaken to inform intervention development and does not evaluate the effectiveness of the resulting intervention on vaccine uptake. Additionally, involvement was shaped by the availability and capacity of partner services, and some perspectives may remain underrepresented. A further limitation is that we did not formally collect demographic information (e.g. ethnicity, nationality) from contributors, which limits our ability to quantify participant diversity beyond the engagement settings themselves.

### 4.6 Implications for Research and Practice

This work demonstrates the feasibility and value of embedding PPPIE within early intervention development in maternal health. It provides a practical example of how involvement and engagement can inform intervention design, particularly in relation to timing, content and accessibility of antenatal vaccine information. Future research should consider how PPPIE can be sustained across intervention implementation and evaluation phases and how learning from involvement can be systematically captured and shared. For practice, this work reinforces the importance of early, accessible information provision and highlights the role of community‐based partnerships in supporting inclusive maternity care.

## 5 Conclusion

This paper describes how PPPIE was embedded within the development of an antenatal vaccination intervention in NI. Input from parents and communities through PPPIE informed the timing, content and presentation of antenatal vaccine information designed for early introduction within the pregnancy care pathway. The approach demonstrates the value of inclusive, early involvement in shaping interventions intended to support informed decision‐making in pregnancy and provides practical learning to inform future maternal health research and practice.

## Author Contributions

**Stephanie Ann McCarron:** conceptualization, investigation, funding acquisition, writing – original draft, methodology, visualization, writing – review and editing, project administration, resources, validation. **Lynsey Patterson:** validation, writing – review and editing, project administration, visualization, supervision, resources, writing – original draft. **Nigel Hart:** conceptualization, investigation, writing – original draft, methodology, validation, visualization, writing – review and editing, project administration, supervision, resources.

## Ethics Statement

Formal research ethics committee approval was not required for this work, as the activities described constitute Patient and Public Involvement and Engagement undertaken to inform intervention development rather than research involving the collection of personal data.

## Conflicts of Interest

The authors declare no conflicts of interest.

## Data Availability

Data sharing is not applicable to this article, as no data sets were generated or analysed during the current study. The work describes Patient and Public Involvement and Engagement (PPIE) activities undertaken to inform intervention development.
